# Associated factors to cervical dentin hypersensitivity in adults: a transversal study

**DOI:** 10.1186/s12903-018-0616-1

**Published:** 2018-09-03

**Authors:** Polyana Matos Alcântara, Nayara Franciele Figueiredo Barroso, Adriana Maria Botelho, Dhelfeson Willya Douglas-de-Oliveira, Patrícia Furtado Gonçalves, Olga Dumont Flecha

**Affiliations:** Dentistry Departament, Federal University of Jequitinhonha and Mucuri Valleys, Rua da Glória, 187, Centro, Diamantina, Minas Gerais 39100-000 Brazil

**Keywords:** Dentin hypersensitivity, Etiology, Diagnosis, Prevalence, Epidemiology

## Abstract

**Background:**

This cross-sectional study aimed to identify the factors associated to the cervical dentin hypersensitivity (DH) in Brazilian adult population.

**Methods:**

Three hundred and eighty patients (67.2% women and 32.8% men) were assessed by questionnaire and thermal test with ice. Participants marked in a visual analogue scale (VAS) the intensity of pain, and a calibrated examiner (ICC 0.990) measured the scores using a caliper. Pain scores above 0.5 mm were considered sensitive teeth. The DH associated factors were investigated by clinical examination. The association between variables was assessed by Spearman correlation and the Chi-square test. Logistic regression was used to determine the variables that predict DH (*p* < 0.05).

**Results:**

The mean age of participants was 24.08 years. In this population, 8958 teeth were evaluated, of those 3037 (33.9%) were diagnosed sensitive. The most prevalent associated factors to DH were abnormal tooth positioning (9.0%), occlusal trauma (6.5%) and gingival recession (5.6%). The erosion predicted significantly the DH on both simple (OR 7.85, *p* < 0.001) and multiple(OR 4.36, *p* < 0.001) analysis.

**Conclusion:**

The exposure of dentinal tubules by erosion is probably the major predictor of dentin hypersensitivity. The healthy tooth is able to protect against DH.

## Background

The dentin hypersensitivity (DH) is defined as an acute pain of short duration due exposure of dentin, in response to stimuli typically evaporative, osmotic (chemical) and tactile, that cannot be associated with any other form of dental defect or disease [1].

The most accepted theory for DH postulates that the fluids of the exposed canaliculi are disturbed by chemical or physical changes. Those changes and movements in the intratubular fluid stimulates baroreceptors which are present in pulp and within the dentin that lead to neural discharge, resulting in painful sensation [2].

The dentin is composed by canaliculi tubules that, under normal conditions, are isolated from the external environment by enamel or cementum. For the development of DH, the dentin, along with their canaliculi, must become exposed to the oral cavity due to gingival recessions and/or loss of enamel/cementum by erosion, abrasion, attrition abfraction, or by incorrect oral hygiene technique [3].

The associated factors that lead to the dentin exposure into the oral cavity and subsequent DH (e.g.*,* gingival recession and non-carious cervical lesions) are also under discussion, in other words, although many have been proposed, the main factors have not been established yet [4, 5].

The DH is a painful condition common in the adult population, its prevalence varies between 3 and 98% [5, 6]. This widely range can occurs due differences in sample, habits, dietaries, eligibility criteria and the diagnostic approaches used for each study [7].

The multifactorial aspect of DH need to be evaluated and to be determined in order to identify the factors causing DH [8]. The treatment of DH should be focused in decreasing or removing it causes and by using DH agents, professionally and/or personally (patient based) methods, to relieve discomfort [7, 9].

There is a lack of data on associated factors relating to this clinical condition among the Brazilian population. Therefore, this cross-sectional study aimed to identify the factors associated to the cervical dentin hypersensitivity.

## Methods

### Study design

This cross-sectional was approved by the Research Ethics Committee of Federal University of Jequitinhonha and Mucuri Valleys (UFVJM) under protocol number 092/2012. It was conducted at the Clinic of Oral Surgery and Periodontics of the Dentistry Department of UFVJM. It was performed according to the Helsinki Declaration of 1975, revised in 2013. All participants of this research have been instructed about the same and signed an informed consent prior the study.

### Sample

The subjects were composed through a stratified sampling among students, professors and employees of UFVJM. It was included those who was older than 18 years, both genders, with good general health and agreed to participate in the research. It were excluded those patients who were undergoing treatment for dental bleaching, in order to avoid overestimation of DH.

To determine the sample size, it was used the calculation for prevalence. It was used statistical significance level at 95% and margin of error set at 5%. Prevalence (33.8%) was obtained in the literature [10]. It resulted in 344 patients to be investigated. It was added 10% to the value to prevent losses. Then, the amount of participants was estimated at 378.

### Dentin hypersensitivity evaluation

One examiner (PMA) was trained and calibrated by test-retest method for use of the caliper. The intraclass correlation coefficient was 0.990.

It was performed clinical examination in order to identify the presence or not of the etiological factors related to DH, and to dismiss pain related to any other tooth pathology. When the DH could not be related to any factor, it was considered “other factors” as etiologic factor. The clinical examination included thermal test which was run on all tooth that were: completelly erupted in the oral cavity, caries-free, restorations free and non-pillars.

In order to identify the DH in each teeth, a cold test was performed using an iced stick on the vestibular surface. The stimulus was maintained until the painful reaction of the volunteer, with a maximum time of 5 s.

After the stimulus was applied, the participants were instructed to mark the pain intensity on a horizontal visual analogue scale (VAS) of 100 mm, where the left and right edges were equivalents to “no pain” and “unbearable pain” respectively. Scores between 0 and 4 mm were considered as no pain; from 5 to 44 mm, mild pain; from 45 to 74 mm, moderate pain; and 75 to 100 mm, severe pain [11]. An analog caliper was used to quantify the values of pain. For a tooth be considered as having DH, it should respond to the cold test with a score equal to or greater than 5 mm.

In cases of gingival recession, its height was measured. It was used Williams periodontal probe, the measurement was taken from the cement-enamel junction to the free gingival margin, in the middle of the vestibular surface.

The participants fulfilled a questionnaire created by the researches in order to identify the oral hygiene habits and previous DH treatment.

### Statistical analysis

The collected data were analyzed by statistical software (Statistical Package for Social Sciences®, IBM Inc., USA) 23.0 version. Descriptive statistical analyses were carried out to obtain frequency, mean and standard deviation. The normality of the data was checked by the Kolmogorov-Sminorv test. The correlation between quantitative variables was done by Spearman correlation. Associations between categorical variables were estimated by the Chi-square test. It was adopted a significance level of 95% (*p* < 0.05).

It was performed logistic regression in order to verify the odds ratio of associated factors that predicts DH. In the non-adjusted regression, it were added the independent variables that obtained a *p*-value less than 0.05 in the Chi-Square test. For the adjusted model, it were selected the variables which had a *p*-value less than 0.05 in the simple regression and the confounding variables (age and sex). The model selection was based on an ascending stepwise procedure including variables to achieve the maximum Hosmer-Lemeshow goodness of fit.

## Results

This research included 125 (32.8%) men and 255 (67.2%) women who contributed with 8958 tooth. The average age of the 380 participants was 24.08 (±7.17) years. Of the participants, 342 (90%) reported being right-handed, 23 (5.7%) left-handers and 15 (4.3%) ambidextrous.

Forty-nine (12.9%) participants had periodontal therapy in the last 6 months and 160 (42.0%) subjects reported to use soft toothbrush. Sixty (15.8%) patients had already treated the DH, and 13 (21.4%) reported no pain relieve (Table 1).

**Table 1:** Oral health care

	n	%
Periodontal therapy (last 6 months)		
Yes	49	12.9
No	331	87.1
Toothbrush bristles		
Extrasoft	6	1.6
Soft	160	42.0
Medium	131	34.6
Hard	23	6.1
Unknown	60	15.8
Dentin hypersensitivity treatment		
Yes	60	15,8
No	320	84,2
Type of DH treatment (*n = 60*)		
In-home	46	76,6
In-office	14	23,3
Results of DH treatment (*n = 60*)		
Temporary relief	36	60,0
Permanent relief	11	18,3
No relief	13	21,4

The amount of 3037 teeth was diagnosed as DH which represents prevalence of 33.9%. The average pain in VAS was 47.3 (±27.5). The Table 2 shows the DH prevalence by teeth group.

**Table 2:** Dentin hypersensitivity prevalence (*n* = 3037 tooth)

Tooth group	n	%
Incisors	1628	53.6
Pre-molars	773	25.5
Canines	525	17.3
Molars	111	3.7

The etiologic factors most prevalent in hypersensitive teeth were abnormal tooth position (9.0%), occlusal trauma (6.5%) and gingival recession (5.6%). Of the hypersensitivity tooth, 2124 (69.9%) were healthy (Table 3).

**Table 3:** Etiologic factors of dentin hypersensitivity (*n = 3037 tooth)*

Etiologic factor	n	%
Abfraction	3	0.1
Atrition	19	0.6
Other factors	41	1.4
Erosion	71	2.3
Bruxism	72	2.4
Inflammation	116	3.8
Gingival recession	170	5.6
Occlusal trauma	196	6.5
Abnormal tooth position	274	9.0
Healthy	2075	69.9

The average of gingival recession height was 1.94 mm (±1.17). There was statistically significant correlation between DH, gingival recession height, and age (Table 4).

**Table 4:** Correlation between dentin hypersensitivity, gingival recession and age

	Dentin hypersensitivity	
	r_s_	*p*
Gingival recession height	0,147	< 0,001
Age	-0,025	0,017

r_s_: Spearman correlation coeficient

There was statistically significant association between DH and: inflammation (*p* = 0.004), attrition (*p* < 0.001), other factors (*p* = 0.019), erosion (*p* < 0.001), bruxism (*p* < 0.001), gingival recession (*p* < 0.001), occlusal trauma (*p* < 0.001), abnormal tooth position (*p* < 0.001) and healthy tooth (*p* < 0.001) (Table 5).

**Table 5:** Association between etiologic factors and dentin hypersensitivity

	Dentin Hypersensitivity		
	Present n (%)	Absent n (%)	*p*-value
Inflammation			
Present	116 (3.8)	161 (2.7)	0.004
Absent	2921 (96.2)	5760 (97.3)	
Abfraction			
Present	3 (0.1)	4 (0.1)	0.617
Absent	3034 (99.9)	5917 (99.9)	
Attrition			
Present	19 (0.6)	6 (0.1)	< 0.001
Absent	3018 (99.4)	5915 (99.9)	
Erosion			
Present	71 (2.3)	18 (0.3)	< 0.001
Absent	2966 (97.7)	5903 (99.7)	
Bruxism			
Present	72 (2.4)	38 (0.6)	< 0.001
Absent	2965 (97.6)	5883 (99.4)	
Gingival recession			
Present	170 (5.6)	94 (1.6)	< 0.001
Absent	2867 (94.4)	5827 (98.4)	
Occlusal trauma			
Present	196 (6.5)	210 (3.5)	< 0.001
Absent	2841 (93.5)	5711 (96.5)	
Abnormal tooth position			
Present	274 (9.0)	374 (6.3)	< 0.001
Absent	2763 (91.0)	5547 (93.7)	
Healthy tooth			
Present	2075 (69.9)	5010 (84.6)	< 0.001
Absent	962 (30.1)	911 (15.4)	
Other factors			
Present	41 (1.4)	49 (0.8)	0.019
Absent	2996 (98.6)	5872 (99.2)	
Quadrant			
First	829 (27.3)	1413 (23.9)	0.107
Second	740 (24.4)	1514 (25.6)	
Third	809 (26.6)	1423 (24.0)	
Fourth	659 (21.7)	1571 (26.5)	

Chi-square test

Erosion, bruxism and gingival recession predicted significantly the dentin hypersensitivity, respectively, on both simple (OR 7.85 [4.67–13.19], *p* < 0.001; OR 3.75 [2.53–5.58], *p* < 0.001 and OR 3.67 [2.84–4.74], *p* < 0.001) and multiple (OR 4.36 [2.56–7.40], *p* < 0.001; 2.34 [1.55–3.51], *p* < 0.001; and OR 2.05 [1.55–2.70], *p* < 0.001) analysis (Table 6). The Hosmer-Lemeshow goodness of fit for the final adjusted model was 0.959.

**Table 6:** Logistic regression of the factors that predicts the dentin hypersensitivity

Variable	Non-adjusted		Adjusted	
	OR (CI 95%)	*p*-value	OR (CI 95%)	*p*-value
Inflammation			–	
Absent	1			
Present	1.42 (1.11–1.81)	0.005		
Abfraction			–	
Absent	1			
Present	1.46 (0.32–6.53)	0.619		
Attrition				
Absent	1		1	
Present	6.20 (2.47–15.56)	< 0.001	3.61 (1.42–9.13)	0.007
Erosion				
Absent	1		1	
Present	7.85 (4.67–13.19)	< 0.001	4.36 (2.56–7.40)	< 0.001
Bruxism				
Absent	1		1	
Present	3.75 (2.53–5.58)	< 0.001	2.34 (1.55–3.51)	< 0.001
Gingival recession				
Absent	1		1	
Present	3.67 (2.84–4.74)	< 0.001	2.05 (1.55–2.70)	< 0.001
Occlusal trauma			–	
Absent	1			
Present	1.87 (1.53–2.29)	< 0.001		
Abnormal tooth position			–	
Absent	1			
Present	1.47 (1.25–1.73)	< 0.001		
Healthy				
No	1		1	
Yes	0.42 (0.38–0.47)	< 0.001	0.53 (0.47–0.60)	< 0.001
Other factors			–	
Absent	1			
Present	1.64 (1.08–2.48)	0.020		
Sex			–	
Male	1			
Female	1.07 (0.98–1.18)	0.118		
Age			–	
≤ 22yo	1			
> 22 yo	1.06 (0.97–1.16)	0.152	–	

*OR* odds ratio. *CI* confidence interval

## Discussion

The dentin hypersensitivity is a common clinical condition which presents several associated factors that should be considered in diagnosis and treatment [12, 13]. The present observational study showed that the mainly DH associated factors involves loss of hard tissue leading to dentin exposure. According to Martens et al. (2013), for dentin exposure it is necessary loss of enamel or cementum exposure [14].

The most prevalent etiological factor was abnormal tooth positioning. The bad positioning, such as crowding of anterior teeth or orthodontic movement, repositions the teeth out of the correct dental arch position. This increases the risk of gingival recessions that can lead to dentin hypersensitivity [8]. Over more, the poorly positioned teeth are difficult to clean up which might causes plaque accumulation and periodontal complications like gingival inflammation and gingival recession. It should be noted that the presence of dental plaque increases the production of acids that demineralize the tooth surface that leads to the exposition of dentinal tubules to oral cavity [15].

The occlusal trauma was considered as etiological factor for DH in 196 teeth. This factor has been cited as responsible for deformation and dental movement, resulting in deflection of tooth structure, broken of enamel crystals in the cervical area and contributing to the exposure of coronal or cervical dentin [8, 16].

An amount of 170 teeth was associated with gingival recession, being the third most prevalent etiological factor in the present study. However, several studies pointed the gingival recession as the main etiologic factor for DH [17, 18, 20]. Once the root and cementum surface is displayed, they are quickly lost by brushing and/or professional cleaning, exposing the dentin tubules [8]. There was a weak statistically significant positive correlation between height of the gingival recession and pain score in the ice test, suggesting that higher the dentin exposed area, greater the response to stimuli and greater the DH pain.

There was a weak statistically significant negative correlation between age and DH. This result disagrees to previous studies that found a greater prevalence of hypersensitivity in the age range of 36–45 [11], 40–49 [21] and 50–59 [10]. However, the present finding corroborates with several researches that found higher prevalence in 20–39 years-old [22, 23]. The differences in the distribution of DH according to age can be due the profile of populations of each study. The negative correlation may also be understand as age-related changes in dentin-pulp complex: the dentin sclerosis and the establishment of secondary and tertiary dentin may be responsible for the decrease in the presence and/or pain level of DH, once the dentin permeability and its the hydraulic conductance is reduced [15, 24].

The erosion was able to predict the DH in both simple and multiple regression, being the factor with biggest odds ratio. The teeth with erosion has odds of 4.36 to have DH in comparison to those with no erosion, independently of other conditions such as age, sex and presence of gingival recession. The present result corroborate the literature which reported that the importance of erosion has become more evident as the main risk factor for DH over recent years [9]. Erosion starts by make softer the plaque-free tooth surface, then, it is followed by constant layer-by-layer dissolution leading to permanent loss of tooth hard tissue with the outer layer soften [12]. This situation allow the dentinal tubules to be patent from the pulp to the oral environment, thereby causing DH.

A great amount of tooth with DH had no associated factors, in these cases, the tooth was considered to be healthy, and the DH was attributed to a failure in cemento-enamel junction (CEJ) [25]. The CEJ morphology may present in three ways: cememtum overlapped the enamel; edge-t-edge relationship between cementum and enamel; and, cementum and enamel fail to meet [26]. This third relationship leads to exposure dentin, which may result in DH.

Also, the great majority of the total investigated teeth was considered healthy and had no DH. The logistic regression showed the healthy tooth as a protective factor against the DH. This finding is in accordance with the hydrodynamic theory [2]. The DH occurs in situations that the dentin become exposed to the oral cavity with open dentinal tubules which provide a direct link between the oral environment and the internal pulp of the tooth [13]. When the exposed dentin contacts with externally stimuli, painful sensations can arise [8]. The protective capable of healthy tooth herein proposed is based on the integrity of the enamel that inhibits the external agents to stimuli the intratubular fluid. However, some tooth may present DH even with no associated factors, in this cases, the DH in healthy tooth can be attributed to a failure in cemento-enamel junction that leads to exposure dentin [25].

The tooth groups most affected by DH teeth were incisors and premolars. Several studies pointed out incisors as most prevalent for presenting a greater wear and loss of enamel and dentin, and for displaying little enamel thick when compared to other tooth groups [8, 27, 28]. In general, teeth most commonly affected are premolars in the adult population [17, 19, 29], due its positioning in the dental arch. They are more likely to be brushed with excessive force, consequently, more disposed to gingival recession and loss of hard tissue [30].

In the future, a growing number of people can be affected by injuries predisposing to dental hypersensitivity due the increasing in longevity and maintenance of teeth in healthy condition [12, 13]. This will bring clinical importance to research new forms of treatment and effective products of easy access. It is of great relevance studies that identify the prevalence and factors associated with this painful condition, since the choice of appropriate treatment depends on a clear conception of the DH by the clinicians [20, 30]. It is important to note that the present data showed that 81.4% of the DH treatment failed which may indicates a recurrent condition. This fact may be explained by the episodic phenome of DH [31, 32].

Hanneet et al. (2016) [8], concluded that it is important to identify the aetiology behind DH, and minimise these contributory factors before formulating treatment strategies. An increase in the understanding of the associate factors involved in dentin hypersensitivity is essential for the development of the clinical management of this condition. Such information could be harvested from epidemiological and interventional studies.

## Conclusion

It can be concluded that incisors are the teeth most affected by DH. The exposure of dentinal tubules by erosion is probably the major predictor of dentin hypersensitivity. The teeth with erosion like to have 4.36 more odds to have DH than teeth without erosion. The healthy tooth is able to protect against DH. Clinician should be able to treat and to manage the DH for a better and lasting relief of pain.

## Abbreviations

DH: Dentin Hypersensitivity
OR: Odds Ratio
UFVJM: Federal University of Jequitinhonha and Mucuri Valleys
VAS: Visual Analogue Scale
